# Physiological Memoir on the Cerebro-Spinal Fluid

**Published:** 1828-11

**Authors:** M. Magendie


### CEREBRO-SPINAL FLUID.

*Physiological Memoir on the Cerebro-Spinal Fluid.* By M. Magendie. Read before the Royal Academy of Science, June 16, 1828; and condensed from the *Journal de Physiologie.*

Since the regard which the ancients entertained for the remains of the dead has given place to an ardent desire to become acquainted with the organization of animal bodies,

Very curious & important anatomical science has been carried to a high degree of perfection by the successive labours of eminent men. Anatomists, who still hope to find some part not observed before, some structure yet undescribed, are fain to proceed with their microscopes in hand. This circumstance alone is sufficient to shew the perfection at which the anatomy of the human body has arrived.

The investigations which I have long pursued with regard to the nervous system have led to my discovering a new element of our constitution: not one of those which requires minute research to be detected: on the contrary, the element of which I speak is so apparent, that it has only escaped heretofore from the belief that no part of the body, however minute, could have eluded the investigation of anatomists.

I have ascertained that there exists in the brain and spinal marrow a liquid, in the midst of which is immersed the brain, spinal marrow, and origins of all the nerves. This liquid, which belongs to the most perfect health, and the quantity of which amounts to several ounces, is too obvious not to have been noticed, and even mentioned, by several authors; but then its presence was attributed either to disease or to changes which had occurred after death. You may conceive my satisfaction on ascertaining so important a fact. A host of conjectures presented themselves to my mind: was this liquid the *animal spirits* of ancient writers?—the *nervous fluid* of which certain physiologists still speak? Doubtless, had the discovery been made fifty years ago, we should have had some hypothesis founded upon it. But such is not now the progress of science: experience is preferred to the most ingenious systems; and some observations on nature and some experiments are all that this memoir will contain.

It was necessary to begin by naming the liquid; for a name is a great matter, even in anatomy. I called it the *cephalo-spinal,* or *cephalo-rachidian,* because it is found both in the head and cavity of the spine. I next tried to determine the exact quantity of it; and I ascertained that, in an adult man of middle stature, and in the enjoyment of all his faculties, there were about three ounces; and in women, under similar circumstances, the quantity is greater: it will be seen by and by that this is no advantage. In old persons the quantity of the liquid is still more considerable, and may extend even to six or seven ounces; but then the faculties both of body and mind are generally much impaired.

The situation occupied by the fluid is worthy of remark. It forms round the brain and spinal marrow a layer which varies in thickness at different points: at the neck it is four or five lines; at the loins it is more than an inch; in the brain generally one or two lines, but in certain structures and certain cases nearly an inch. Do not these facts militate strongly against a famous system, in which it is pretended to determine the most minute circumstances concerning the volume and conformation of the brain by the shape of the skull. If there exists, as cannot be doubted, a layer of fluid between the cranium and the brain, and if this layer may have several lines in thickness, how can we judge of the dimensions of the brain by those of the cranium? and how be sure that the elevations or depressions of the surface of the head correspond to those of the brain?

The study of the portion of the fluid which covers the brain led me to a singular and very unexpected fact with regard to the volume of this organ.

We look upon the dimensions of the brain as not subject to variation, because we think that it fills exactly the cavity of the cranium, and because we do not see the head become emaciated or increased in size with the other parts of the body: but nothing is less correct. I ascertained that the brain follows the other organs with regard to the change of its volume.

In all diseases of a certain duration, when the body wastes much, the brain undergoes a similar diminution, and the convalescent who can scarcely walk, and who attributes his weakness to the almost entire disappearance of the muscles of his limbs, might with as much reason attribute his moral weakness to the diminution in the size of the brain. I have ascertained, besides, that, in proportion as the wasted limbs regain their former dimensions, the brain also recovers that which it had lost. Thus it appears that one of the uses of the cephalo-spinal liquid is to replace the brain as often as it diminishes in actual volume. It fulfils the same office in instances of partial diminution, as I was able to determine in individuals who, during many years of their life, had had the arm or leg contracted and immovable. In this case a fifth or fourth part of one cerebral lobe had disappeared; a large depression was found on the surface of the organ, and this was occupied by the cerebro-spinal liquid, so that the cranium was always full.

After having made out the physical uses of the liquid, I wished to ascertain whether it exercised any influence on life. This could only be done by experiment on the lower animals, which have the cerebro-spinal liquid, though in much smaller proportion than man. My first trial was upon a fierce old fox, who was not at all anxious to contribute to the advancement of science. By means of a little puncture made in the neck, he lost all the cerebro-spinal liquid in a few minutes. The effect was very striking: this animal, which had been ferocious but a moment before, became calm all at once; he no longer attempted to bite, and indeed scarcely moved. Seeing him in this disposition, I made him be set at liberty; but he lay down on the spot, and did not stir till next morning; he then attempted to get up, and in the course of the day made several steps with some confidence. At the end of thirty-six hours he again attempted to bite, and tried to escape. I then made a fresh puncture in his neck, and from the result I was satisfied that the cerebro-spinal liquid had been completely renewed again.

These inquiries led me to examine, with more attention than I had previously done, a disease of very young infants, in which a pouch filled with water exists at the lower part of the spine, at the place where the natural liquid is in large quantity; and I discovered that what we regard as a morbid product is nothing more than the natural liquid, which has distended its envelopes, and formed a hernia externally. When this bag happens to burst, the liquid escapes, and death speedily follows: probably because, the aperture remaining open, the fluid cannot again collect, and protect the brain and spinal marrow by its presence. Thus it appears that in man, as in the lower animals, the contact of this liquid with the surface of the brain is of great importance to the perfection of the nervous functions, and even to life.

But is it merely as a fluid that this is of so much use? or are the functions at all dependent upon its chemical constitution? To determine this question I made an experiment, in which, after having exhausted the cerebro-spinal liquid of an animal, I supplied its place with an equal quantity of distilled water at the same temperature; and I found, with surprise, that the animal became extremely agitated: its movements were perverted; it appeared to have entirely lost its usual instincts and habits. All these phenomena ceased when I allowed the water to escape. To judge if the temperature had an effect on the nervous functions, after having allowed the liquid which I had previously extracted from the animal to become cold, I reintroduced it into the cavity which it had occupied. Immediately the animal was seized with general trembling, analogous to what succeeds intermittent fevers. It is, therefore, not impossible that this experiment may throw some light on the cause of the cold stage in fever.

I conclude, from the facts and experiments which I have detailed, and from many others already published, that the cephalo-spinal liquid influences the functions of the nervous system, first, by its cóntact with the surface of the brain and spinal marrow; secondly, by its chemical composition; and thirdly, by its temperature; and thus that this liquid must be ranked along with the blood, lymph, &c. from its utility in the animal economy.

But I had a much more important object than that which we have considered: I had to study the influence of this liquid upon the intellectual faculties of man. That I may be the better understood, it is necessary to say a few words on the formation of the brain. This is divided into two portions, —one large, and occupying the upper part of the cranium, viz. the *brain* proper; the other small, and placed beneath, viz. the *cerebellum*. The exterior of the brain presents a great number of rounded protuberances, varying in different individuals, and separated by furrows. Numerous cavities are formed in the centre of the cerebrum. It is there most probably that some of the mysteries of nervous actions and intellect are accomplished. Can it be believed that these cavities, rendered so important by the phenomena there produced, have been, and still are, denominated *ventricles*—little bellies! Is it not high time to discard this frivolous appellation from the language of anatomy? However this may be, the nomenclature of the parts contained within the cavities of the brain offer this remarkable circumstance, that many of them have names indicative of hydraulic uses: thus we have the *aqueduct,* the *funnel,* the *valve,* and even the *bridge.*

Most of these names have descended to us from distant periods, and we are accustomed to look upon them as the remains of some ancient system which has crumbled beneath the weight of time. The old physicians believed that the ventricles of the brain were filled with water, which in certain cases escaped by the nose; a belief which passed to the vulgar, among whom we still meet with it. These ideas are looked upon as erroneous by modern anatomists, according to whom the ventricles of the brain, in its healthy condition, do not contain any water, but an invisible vapour, which they have represented as the immaterial spirit presiding over the acts of intelligence. Nevertheless, when we open the brain, we almost always find the ventricles filled with a limpid fluid; but the present doctrine regards this as the product of the disease causing death.

Having acquired the data which I have already mentioned, I have been led to think that the water which is so frequently found in the cerebral cavities might be the same which is found on the surface of the brain; from which it would follow that its presence in the ventricles was natural, as held by the ancients, and not a morbid product, as is at present supposed.

It will be perceived that, in order to confirm this idea, it was absolutely necessary that there should exist an opening by which a communication might be established between the exterior of the organ and its internal cavities; and yet no such opening was known. I did not, however, despair; and after some researches, made at the termination of various diseases, I at last found an aperture, two or three lines in diameter, completely hid by a lobe of the cerebellum, and forming a true *entrance to the cavities of the brain.*

This fact once established, it became mechanically necessary that the cerebro-spinal liquid should enter into the cavities of the brain, and fill them; for they communicate with each other. I had no difficulty in verifying this inference in the bodies of persons destroyed by accidents, and which, in fact, shewed me the liquid filling the cerebral cavities.

This discovery explained the hydraulic nomenclature of which I have spoken. I perceived that these pretended notions of ancient doctrines were simply the figurative but just designation of an assemblage of organs in full activity. In fact, the valvula Vieusseni of the cerebellum fulfils, to a certain extent, the office of a *valve*; the *aqueduct* has actually the functions which the name implies, as it transports the liquid from the fourth to the third ventricle; the infundibulum, or *funnel*, carries it to the pituitary gland; and, lastly, the pons, or *bridge*, is really an arcade placed transversely in the direction which the fluid observes: it is situated not over, but under, the current which it traverses; and, to give an idea of it, I cannot do better than call to mind the tunnel which is now in progress under the Thames!

This, then, is a complete explanation of the hydraulic apparatus presented by the brain. Without being an exclusive admirer of ancient times, I must remark that, in this instance at least, our predecessors had observed with more accuracy than we have done. Modern anatomists, however, have this merit, that they respected the names, although they regarded them as false; and in this they were wise, as people sometimes are, without suspecting it.

The liquid which fills this cavity is not in repose. On the contrary, it undergoes constant agitation, by the effect of a kind of flux and reflux resulting from respiration. Thus, at the moment we inhale, the liquid flows out from the cerebral cavities, and passes into the spinal canal; while, on the other hand, at the moment we expire, the liquid re-enters those cavities, and flowing by the conduits above mentioned, particularly the *aqueduct*, which gives passage to the fluid, now in one direction, and now in the opposite.

The mechanical cause of this flux and reflux is very simple: it depends upon the alternating turgescence of the veins of the spine under the influence of respiration. This movement of the liquid is arrested, or much retarded, by compressing the abdomen. We may remark, that this is one of the effects of girdles, and serves to explain how their use becomes dangerous, or even insupportable, when the pressure is too great.

In studying the passage of the fluid by the aqueduct, I believe I have discovered the probable use of the pineal gland. I look upon it as a stopper destined to open and shut the aqueduct, over the anterior opening of which it is situated. Two large veins are placed and fixed upon the gland: these vary in size. Sometimes they swell greatly, and at others are nearly empty. It is inevitable, from the relative position of the parts, that the moment the veins swell they must press down the pineal gland, and this cannot yield nor descend without shutting the entrance of the aqueduct to a greater or less extent. Now, as one of the constant effects of exertions, anger, and all violent passions, is to swell the veins of the head, and particularly those which press upon the pineal gland, it follows that, in these different conditions, the entrance of the fluid into the ventricles is intercepted, or at all events impeded. The use, then, (or, more correctly, one of the uses,) of the pineal gland, would appear to be that of regulating mechanically the flow of the cerebro-spinal liquid through the aqueduct.

What influence has this fluid on the intellectual faculties? With a view of ascertaining this, I have endeavoured to determine the quantity in the brains of sane persons, of maniacs, and idiots. Idiots have a considerable quantity: it occupies the surface of the brain, and there forms a thick layer; it distends the cerebral cavities, and displaces all the parts found there, particularly the pineal gland, which, driven from its natural position, can no longer fulfil the functions which I attribute to it. The aqueduct likewise presents a considerable enlargement. In these cases we find six or seven ounces of the cerebro-spinal fluid; and it is the same in the fatuity of elderly persons.

Maniacs also have a large quantity of liquid, but it does not accumulate at the surface of the brain : whatever be the nature of the insanity, the ventricles are always much distended.

The brains of persons gifted with reason up to the period of their death generally contain less than an ounce of fluid in the ventricles; so that it is easy in this way to distinguish the brain of a madman, a fool, or a sane person. It would appear that the development of the mental faculties is in the inverse ratio of the quantity of the cerebro-spinal liquid; and this, to a certain extent, is easily understood, as the volume of the fluid can only increase at the expense of the solid mass of the brain.

I may add, that it is not merely necessary that the liquid shall not be too abundant, but also that its movement be free. I lately found, in the brain of an old opera singer, who died idiotic at the Salpetriere, an obliteration of the opening by which the liquid enters the ventricles; and, as there was no other disorganization, I am inclined to think that this obliteration was the cause of the malady.

---

